# Motorless cadence control of standard and low duty cycle-patterned neural stimulation intensity extends muscle-driven cycling output after paralysis

**DOI:** 10.1186/s12984-022-01064-w

**Published:** 2022-08-09

**Authors:** Kristen Gelenitis, Kevin Foglyano, Lisa Lombardo, John McDaniel, Ronald Triolo

**Affiliations:** 1 Louis Stokes Cleveland VA Medical Center, 10701 East Blvd, Cleveland, OH 44106 USA; 2 Kent State University, 800 E Summit St, Kent, OH 44240 USA; 3 Case Western Reserve University, 10900 Euclid Ave, Cleveland, OH 44106 USA

**Keywords:** Paralysis, Spinal cord injury, Exercise, Neural stimulation, Cycling, Feedback control

## Abstract

**Background:**

Stimulation-driven exercise is often limited by rapid fatigue of the activated muscles. Selective neural stimulation patterns that decrease activated fiber overlap and/or duty cycle improve cycling exercise duration and intensity. However, unequal outputs from independently activated fiber populations may cause large discrepancies in power production and crank angle velocity among pedal revolutions. Enforcing a constant cadence through feedback control of stimulus levels may address this issue and further improve endurance by targeting a submaximal but higher than steady-state exercise intensity.

**Methods:**

Seven participants with paralysis cycled using standard cadence-controlled stimulation (S-Cont). Four of those participants also cycled with a low duty cycle (carousel) cadence-controlled stimulation scheme (C-Cont). S-Cont and C-Cont patterns were compared with conventional maximal stimulation (S-Max). Outcome measures include total work (W), end power (P_end_), power fluctuation (PFI), charge accumulation (Q) and efficiency (η). Physiological measurements of muscle oxygenation (SmO_2_) and heart rate were also collected with select participants.

**Results:**

At least one cadence-controlled stimulation pattern (S-Cont or C-Cont) improved P_end_ over S-Max in all participants and increased W in three participants. Both controlled patterns increased Q and η and reduced PFI compared with S-Max and prior open-loop studies. S-Cont stimulation also delayed declines in SmO2 and increased heart rate in one participant compared with S-Max.

**Conclusions:**

Cadence-controlled selective stimulation improves cycling endurance and increases efficiency over conventional stimulation by incorporating fiber groups only as needed to maintain a desired exercise intensity. Closed-loop carousel stimulation also successfully reduces power fluctuations relative to previous open-loop efforts, which will enable neuroprosthesis recipients to better take advantage of duty cycle reducing patterns.

## Background

People with spinal cord injury (SCI) or other neuromuscular disorders are at high risk for secondary health issues due to immobility from lost volitional muscle control [1, 2]. Electrically-induced cycling engages paralyzed musculature in exercise and prevents or mitigates negative health consequences such as muscle atrophy, poor circulation, increased fat mass, and reduced quality of life [3–6]. However, such improvements often develop slowly as rapid muscle fatigue is common with these systems and greatly reduces sustained exercise intensity and endurance within a single session [7]. Additionally, improvements in physiological factors that are load dependent, such as bone density, are not yet well established because the limited sustained force production prevents prolonged cycling against sufficient resistances [8–10].

Recent studies have shown significant improvements in exercise ability with selective stimulation strategies [11–13]. Asynchronous or interleaved stimulation has been shown to increase sustained force and delay induced muscle fatigue during dynamic knee extension for participants with SCI [14–16]. Additionally, our group demonstrated that reducing either the duty cycle of the activated knee extensor musculature or the overlap of electric fields from nearby stimulating electrodes significantly increases work performed and power maintained over conventional, supramaximal stimulation within a cycling exercise session [13]. Submaximal levels of stimulus delivered through independent electrode contacts can activate non-overlapping motor unit pools (MUPs) and avoid over-stimulating the common fibers that would otherwise be within the overlapping electric field regions. Duty cycles can be reduced through “carousel” stimulation schemes that rotate activation among the independent knee extensor fiber groups by stimulating through a different contact each pedal rotation. Longer recovery periods between successive contractions of the same group of fibers improve power maintenance and increase total work performed in low duty cycle patterns. While these strategies were largely successful in open-loop implementation [13], significant power output fluctuations sometimes occurred with carousel patterns due to variations in the stimulated strength of each independent MUP. These fluctuations due to uneven force production among independent fiber groups caused pedal strokes to vary in strength and speed.

Another strategy that may address the variability in contraction strength and further improve exercise performance after paralysis is closed-loop control of the stimulation intensity to maintain a consistent, but submaximal level of cycling power. Open-loop cycling programs employ preset levels of stimulation throughout the exercise. Unlike volitional exercise in able-bodied individuals that stochastically activates only the motor units required to maintain a desired intensity [17], such approaches continuously activate and subsequently fatigue all recruited motor units from the onset of exercise. Instead, modulating stimulation levels from initially low overlap values with feedback control has the potential to target submaximal exercise intensities that result in a greater overall steady state power output. While this would result in a lower initial peak intensity, adjusting stimulation as needed to recruit not-yet-fatigued fibers could maintain a mid-level cycling power for longer and ultimately improve endurance and produce more work within an exercise session. This may also address the power fluctuation issues when combined with duty cycle reducing stimulation patterns by ensuring each fiber group produced similar outputs to match a steady target value when active.

The present study implemented closed-loop control of neural stimulation levels on a motorless recumbent trike. We investigated the relative performances of open-loop cycling with a fixed knee extensor stimulation intensity, closed-loop modulation of synchronous activation of all available knee extensor MUPs, and closed-loop carousel stimulation that modulated activation of a different independent MUP each successive pedal stroke in participants with chronic paralysis due to SCI or other upper motor neuron dysfunction. We hypothesized that closed-loop control would improve endurance in terms of end power output and work performed, reduce power fluctuations, and increase efficiency in terms of output per unit charge within an exercise session over conventional open-loop stimulation. We further hypothesized that functional improvements would correlate with positive impacts on physiological responses to exercise.

## Methods

### Experimental setup and protocol

**Fig. 1:**
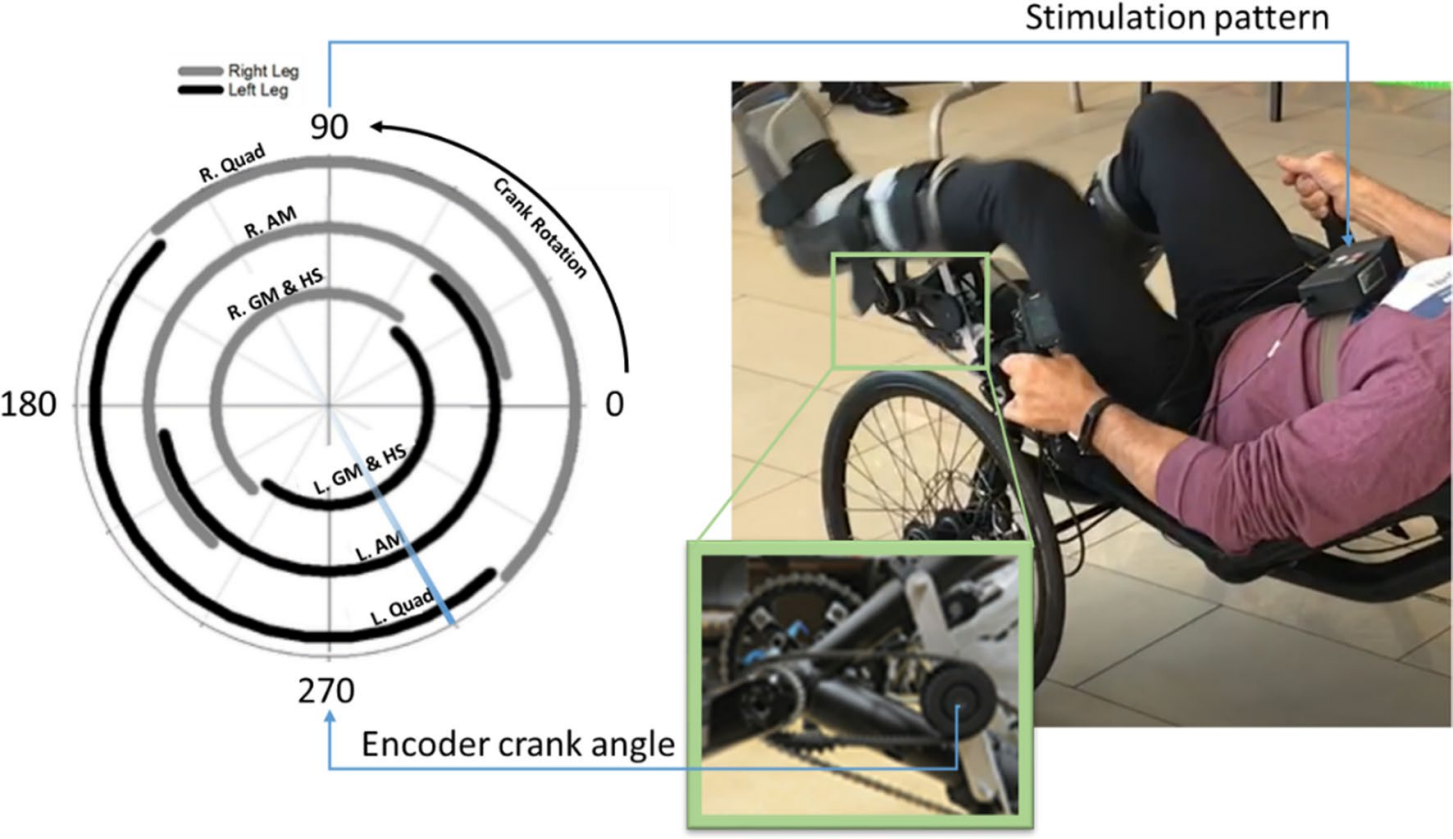
Neural stimulation-driven cycling exercise setup

Seven participants with paralysis performed cycling trials. Each had previously received an implanted neural stimulation system to activate the otherwise paralyzed musculature of the trunk and lower extremities. The implanted systems with a global reference and stimulating electrode variations (single contact epimysial, single or four contact spiral cuffs, and eight contact C-FINEs) used in biking are described elsewhere [13]. A summary of paralysis classifications and relevant implanted electrodes is provided in Table 1. All participants, regardless of classification, exhibited no volitional control over the muscles activated with stimulation during cycling, with MRC manual muscle test scores of zero. Participants were seated on a recumbent bike (Catrike, Orlando, FL) with their legs secured in custom orthotics affixed to the pedals (Fig. 1). A crank angle encoder (US Digital, Inc.) relayed instantaneous pedal position to an external control unit (ECU) running a custom stimulation model designed in Simulink (Mathworks, Natick, MA, USA). Within the stimulation model, pedal positions were mapped to the appropriate muscle activations and corresponding stimulus patterns that produced smooth cycling based on able bodied and surface stimulation cycling literature [18, 19]. Muscle activation timing patterns were further customized heuristically with participants in the loop to adjust for differences in the participants’ muscle function and implanted system capabilities. The ECU relayed the desired stimulus parameters (pulse amplitude, pulse duration and stimulus channel) based on crank angle to the implanted pulse generator via an external radiofrequency coil. The pulse generator then delivered stimulating current through various implanted electrode contacts on or near the peripheral nerves that activated the paralyzed musculature and induced the cycling movement.

**Table 1:** Summary of participants and corresponding implanted knee extensor-activating electrode details

Participant	Paralysis cause/classification	Knee extensor stimulation	Independent knee extensor contacts/(total available independent contacts) per leg
P01*	C7 AIS-B SCI	Proximal femoral spiral cuffs + Vastus Lateralis epimysials	2/(4) + 1/(1)
P02*	T10 AIS-A SCI	Proximal femoral C-FINEs	3/ (8)
P03*	C5 AIS-C SCI	Proximal femoral C-FINEs	3/(8)
P04*	T11 AIS-B SCI	Proximal femoral spiral cuff	3/(4)
P05	T4 AIS-B SCI	Proximal femoral spiral cuffs	1/(1)
P06	T3 AIS-A SCI	Proximal femoral spiral cuffs	1/(1)
P07	Adult Onset Adrenoleukodystrophy	Proximal femoral spiral cuffs	1/(1)

Asterisks (*) indicate participants with multiple independent knee extensor stimulation channels enabling cycling with carousel cadence-controlled patterns

Electrode contacts that activate the quadriceps, hamstrings, hip adductors, and gluteal muscles can all be involved in cycling exercise patterns. For this study, only stimulation delivered through contacts that activated the quadriceps (knee extensors) muscle groups varied among stimulation conditions. All other muscle groups activated during the cycling activity received maximum stimulation levels in each condition. The conventional open-loop stimulation pattern, Standard Max (S-Max), delivered supramaximal stimuli through all knee extensor-activating electrode contacts each pedal rotation. Standard Controlled (S-Cont) stimulation also delivered stimulation through all knee extensor contacts each pedal rotation, but simultaneously modulated pulse width (PW) delivered through those contacts on a given leg instead of using a fixed supramaximal value. Carousel Controlled (C-Cont) stimulation modulated stimulation PW through a different knee extensor contact for each pedal stroke, thus reducing the duty cycle while controlling the output of each independently activated fiber group. Only participants with multiple independently controlled knee-extensor activating contacts (P01–P04) were able to cycle with the C-Cont pattern (Table 1). Low overlap among the fiber groups activated by each contact during carousel stimulation at submaximal PWs was established for P01 and P02 in a prior study through moment summation tests [13]. Overlap was not formally assessed for P03 and P04 due to availability constraints.

**Fig. 2:**
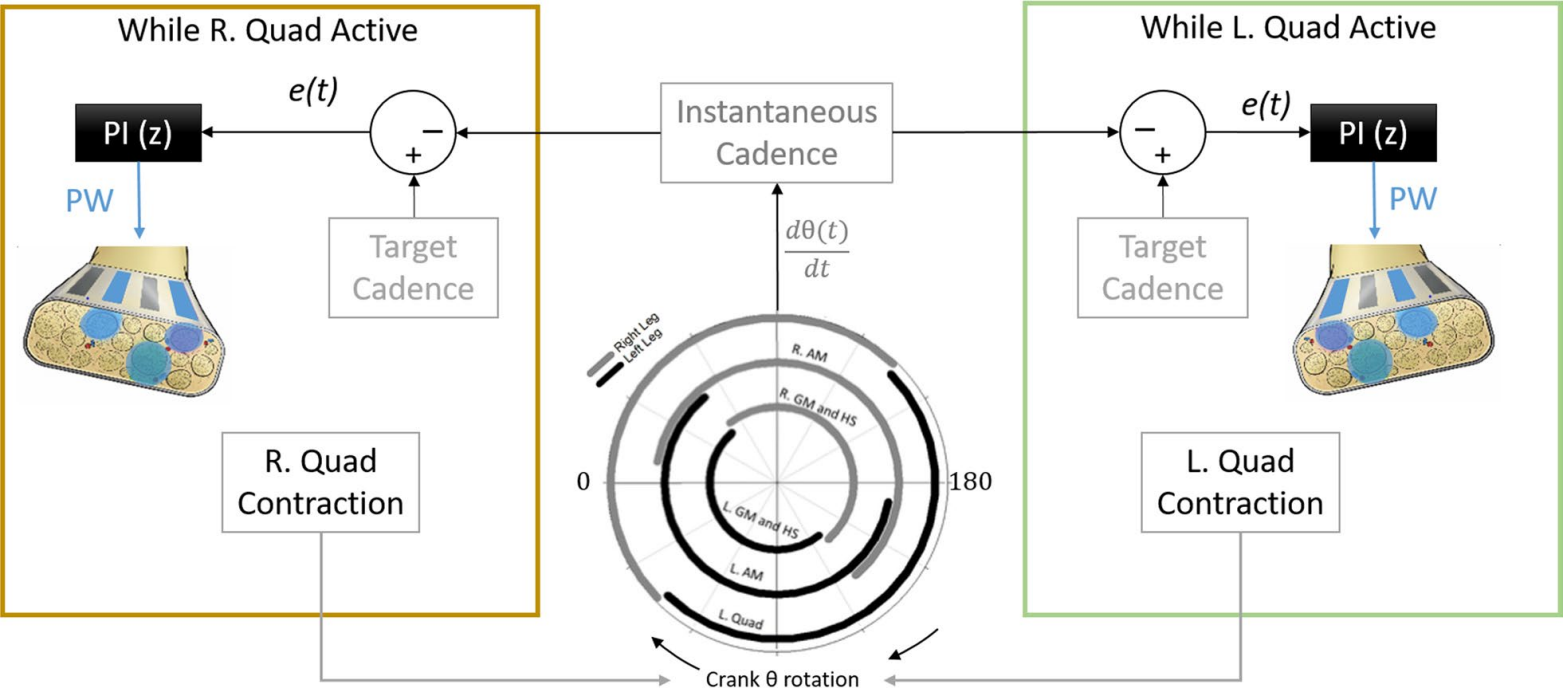
Standard Controlled (S-Cont) stimulation schematic. Instantaneous cadence is calculated from moving-average filtered time derivative of the crank angle and compared against a target cadence. An error *e(t)* between target and instantaneous cadence drives one PI controller per leg to adjust the PW delivered through all knee extensor-activating contacts during the respective left and right active periods of quadriceps activity during the stroke cycle

**Fig. 3:**
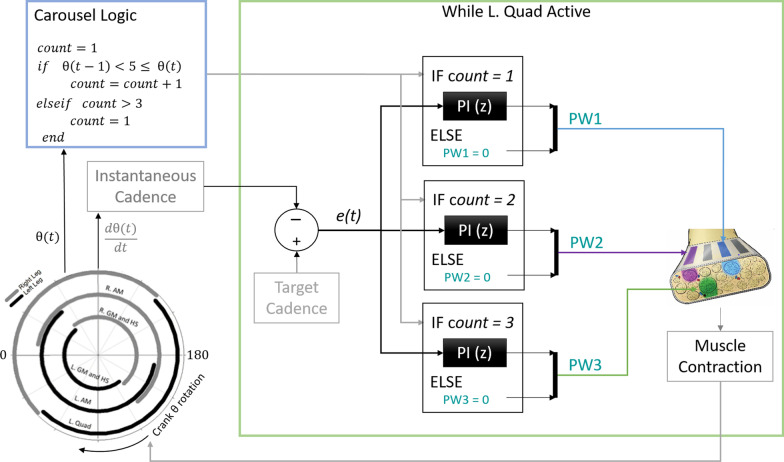
Carousel Controlled stimulation schematic for the left quadriceps (green box). Carousel logic (blue box) detects the passing of an angle (θ = 5) outside of the L. Quad active region and switches the stimulating contact and thus fiber group active in the next contraction. Instantaneous cadence and resulting errors are calculated as in the Standard Controller. Each independent contact is driven by its own independent PI controller, enabling different PW outputs to be delivered through the different contacts when active. When not active, contacts receive a PW of zero and thus do not contribute to the pedal stroke. This logic is repeated for the right quadriceps, using a different crank angle (θ = 180) as the contact switching signal

Both controlled patterns began at submaximal stimulus levels for all participants. Differences between actual cycling cadence and target cadence created an error signal *e(t)* that drove proportional-integral (PI) controller(s) to adjust PW delivered by the active contact(s) (Figs. 2, 3). Error calculations were recomputed every stimulation period (0.04 ms, 25 Hz) such that PW values were adjusted for each pulse within the knee extensor phase of the pedal stroke. P and I gains (0.37 ± 0.12 and 0.54 ± 0.16, respectively) were initially set according to those found to best track a sinusoidal target output during isometric studies [20] and further heuristically adjusted as necessary to produce smooth but responsive cycling. Instantaneous cadence was calculated in the stimulation model as the rate of change of the crank angle. Because cadence is proportional to different power outputs for each gear on the drivetrain, the participant remained in the same gear throughout these cadence-controlled trials to ensure actual power was in the desired mid-level range. Controlled trial fixed gears and target cadences were chosen such that corresponding target power outputs would be between peak and steady state powers produced with S-Max stimulation. Stimulus pulse amplitude (PA) remained fixed at the lowest value that produced a full range of contraction strength from just noticeable to maximal contraction over the available PW range (PA = 0.8 or 2.1 mA for all quadriceps-activating contacts).

Each experimental session began with a short warm-up trial with S-Max stimulation to reduce any spasms or initial tone, followed by an S-Max trial to assess baseline performance. Subsequent trials alternated between a controlled condition and S-Max with breaks at least double the cycling duration between trials to prevent cumulative fatigue. Exercise trial durations were 300, 150, 90, 90, 180, 150, and 120 s for participants 1–7, respectively, and were determined based on the amount of time the participant could continuously cycle with S-Max. Though trial durations varied by participants based on ability level, trial durations were kept consistent across simulation conditions for each participant.

### Outcome measures

A Garmin Edge bike computer (Garmin Ltd., Olathe, KS) communicating with Quarq DZero power crank arms (SRAM LLC, Chicago, IL, USA) provided functional cycling outcome measures. Total work (W), calculated as cycling power output integrated over trial duration, provided a measure of exercise intensity. End power (P_end_), the average power output over the final third of each trial, provided a measure of steady state power maintenance and thus endurance. A power fluctuation index (PFI) was calculated for each trial as the mean ratio of peak-to-peak power relative to the detrended average power over each 6 s window to encompass several full pedal revolutions. A lower PFI indicates a more consistent power output and smoother ride. Root-mean-squared error (RMSE) was calculated for controlled conditions to determine how well a target cadence was maintained by a given controller configuration. RMSE is calculated only for the portion of a trial up to Time on Target (T_target_), taken as the final timepoint where target cadence was achieved, in order to reflect controller performance only while dynamically adjusting PW, prior to being unable to compensate for fatigue once reaching hardware-limited maximum PW values. Beyond T_target_, declines in actual cadences reflect limitations in muscle output capabilities, not active controller performance.

Initially, trials were performed in the Motion Study Laboratory at the Louis Stokes Cleveland VA Medical Center. In-laboratory trials ran custom Simulink cycling models through MATLAB real-time xPC target, from which controller PW outputs could be accessed and analyzed. Time to Max PW (T_maxPW_) was calculated for controlled conditions as the time at which stimulation through all controlled contacts reached stimulator maximum. Total charge injection (Q) was calculated:

$$\mathrm{Q}=\sum_{n=1}^{c}\left[\left(\int {PW}_{n}\right)x {PA}_{n}\right],$$

where c is the combined number of knee-extensor activating contacts on the left and right legs. Differences in controller efficiency (Δη) between a controlled condition and S-Max was then calculated as:

$$\Delta \eta = \frac{{W}_{Cont}}{{Q}_{Cont}}- \frac{{W}_{S-Max}}{{Q}_{S-Max}}.$$

Higher η indicates that a condition produced more work per unit of charge injected, and thus exhibited higher efficiency. Due to the COVID-19 pandemic and subsequent pause on in-person research, several cycling sessions were performed remotely by participants who already had recumbent cycling setups in their own homes. In these cases, stimulation models were compiled into standalone ECUs. This enabled successful deployment and testing of various controller patterns remotely, but did not allow collection of controller output due to ECU storage limitations, thus preventing analysis of T_maxPW_, Q, and η in these cases.

Two physiological metrics were also assessed with select participants. MOXY muscle oxygenation monitors (Fortiori Design, LLC, Hutchinson, MN, USA) non-invasively measured the muscle oxygen saturation (SmO_2_) of various activated heads of the quadriceps in four participants. SmO_2_ is the ratio of oxygenated hemoglobin and myoglobin to total hemoglobin and myoglobin in the underlying muscle tissue. Declining SmO_2_ values indicate the muscle fibers are utilizing oxygen faster than they are being supplied, and that an exercise intensity is likely not sustainable under current conditions. Lastly, heart rate was monitored during select trials with one participant using a Vivosmart (Garmin Ltd., Olathe, KS) wrist-worn activity tracker to determine if any resulting functional improvements in cadence-controlled cycling performance would be sufficient to evoke corresponding cardiovascular responses.

### Statistical analyses

Participants completed at least two (median: 4, range: 2–12) trials of a cadence-controlled stimulation condition and a corresponding number of conventional stimulation trials for comparison. Number of trials completed depended on an individual participant’s availability and ability level. Within-subjects statistical analyses were performed to assess outcome differences between conventional and a cadence-controlled stimulation condition for a given participant. A within-subjects approach was used instead of group statistical analyses due to heterogenous paralysis causes and levels, stimulation system configurations, baseline strengths, and number of trials performed [21]. Mann–Whitney nonparametric tests were applied to W and P_end_ data as results were independent, homogenous (Levene’s test for equal variances p > 0.05), but non-normal (Shapiro-Wilks tests for normality p < 0.05). PFI and heart rate data sets were compared using Welch’s ANOVAs followed by post-hoc Dunn’s tests.

## Results

### Functional outcomes: total work, end power, and power fluctuation

**Fig. 4:**
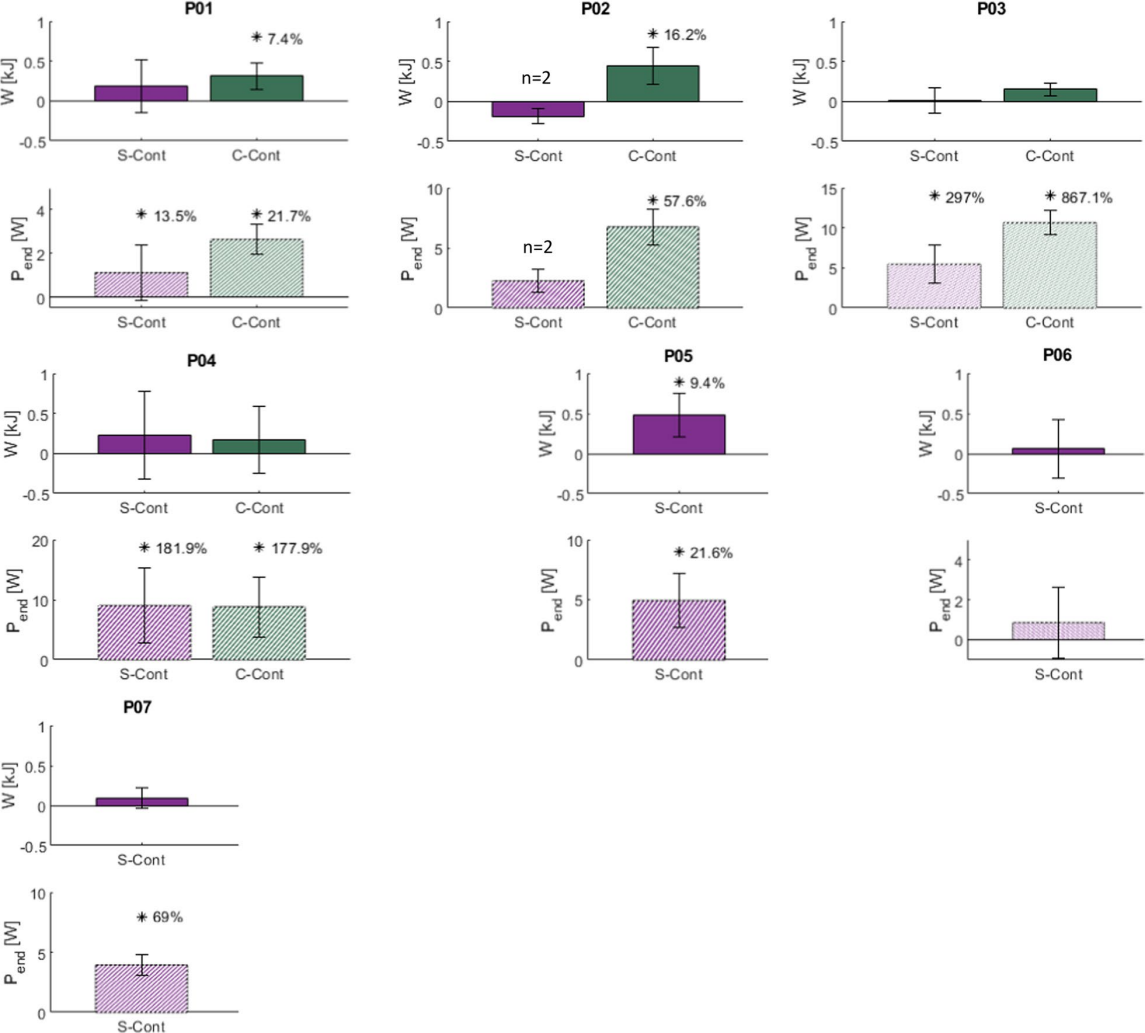
Difference in W and P_end_ between controlled conditions and S-Max stimulation trials. Positive differences indicate improved outcomes compared with conventional, open-loop cycling. Percent improvement is given for differences with statistical significance (*p < 0.05). Note participant P02 only completed 2 trials of the S-Cont condition due to time constraints. All other participants completed at least three trials of cadence-controlled conditions and a corresponding number of S-Max trials

Differences in W and P_end_ outcome measures between trials of a given controlled test condition and S-Max stimulation trials are presented in Fig. 4. Positive differences indicate a controlled condition improved total work performed or power maintained over conventional stimulation within the same trial duration. Increased ΔW suggests a more intense bout of exercise. Increased ΔP_end_ demonstrates greater achieved endurance as higher power was maintained through the end of the trial. In general, controlled conditions were found to improve W and P_end_ over conventional stimulation.

S-Cont significantly increased P_end_ in five out of the seven participants tested (P01: 13.5%, P03: 297%, P04: 182%, P05: 21.6%, and P07: 69%), but produced a significant improvement in W in only one participant (P05: 9.4%). C-Cont stimulation significantly increased P_end_ in all four participants tested (P01: 21.7%, P02: 57.6%, P03: 867.1%, P04: 178%), and significantly increased W for two of those participants (P01: 7.4% and P02: 16.2%).

**Fig. 5:**
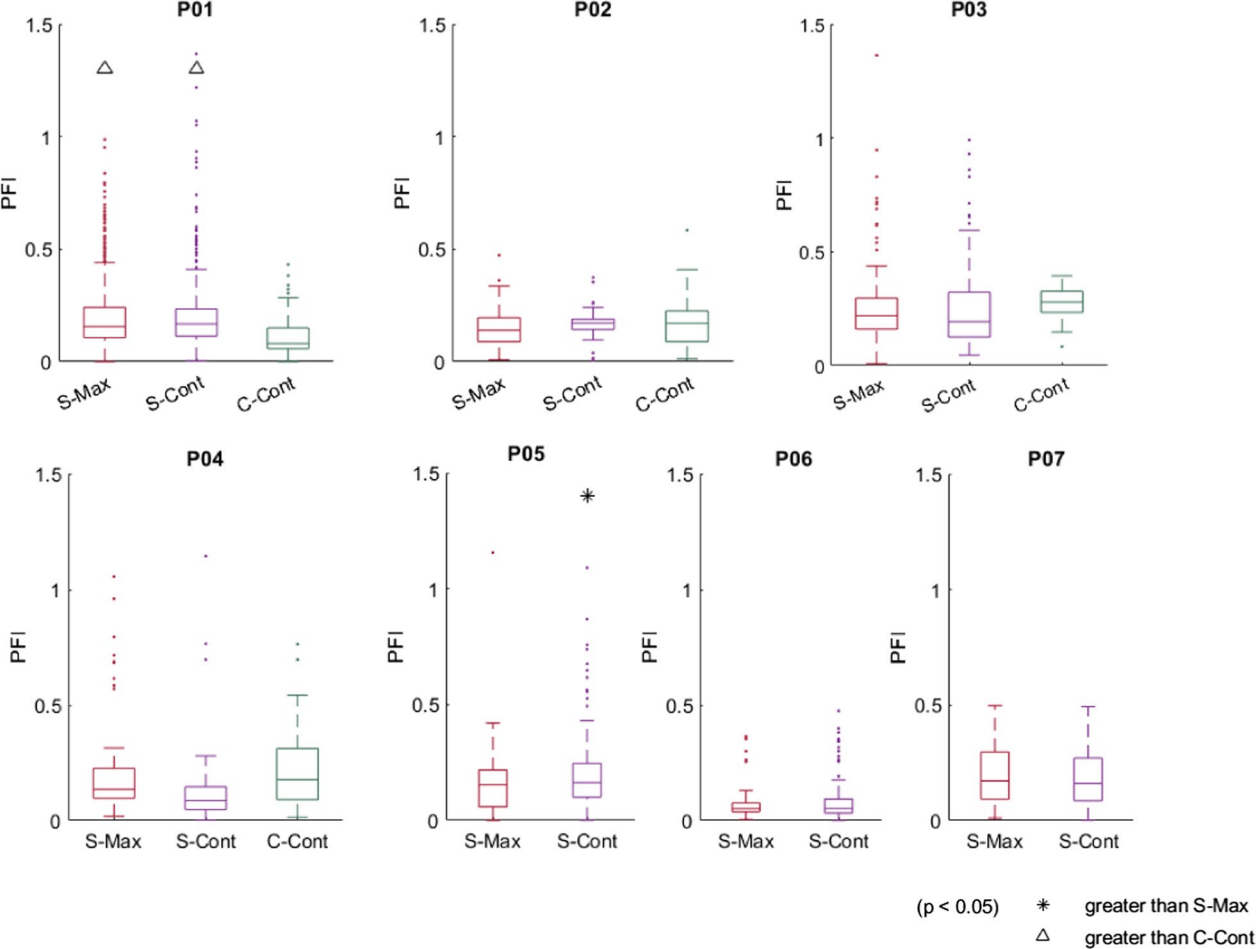
Power fluctuation indices (PFI) for conventional and cadence-controlled stimulation conditions. Lower PFI indicates a smoother, more stable ride

PFIs resulting from S-Max and cadence-controlled conditions are presented in Fig. 5. C-Cont stimulation significantly reduced PFI to the point of no significant difference with S-Max in three of four participants tested here, as well as relative to open-loop low duty cycle approaches from prior studies [13]. In the fourth participant, C-Cont decreased PFI compared with both S-Max and S-Cont (median = 0.08 vs. 0.16 and 0.17, respectively). S-Cont increased PFI over S-Max with statistical significance in just one participant, although absolute differences were quite small (median = 0.17 and 0.15).

### Controller performance: RMSE, charge accumulation, and efficiency

Controller performance metrics are presented in Table 2. Target cadences ranged from 25 to 52 rpm and were based on participant S-Max cycling ability in an exercise session, and thus may vary between and within controlled conditions for each participant. Available T_maxPW_ data closely matched T_target_ within each participant for a given controlled condition, demonstrating target cadences were maintained while controllers modulated PW below maximum. Average RMSE and RMSE as a percent of target cadence (RMSE %) ranged from 1.1 to 3.7 rpm and 3.4–10.5%, respectively, indicating good controller tracking performance prior to steady cadence decline due to advanced fatigue.

**Table 2:** Controller performance for controlled stimulation conditions

Participant	Controller type	Target cadence [rpm]	T_maxPW_ [s]	T_target_ [s]	RMSE [rpm]	RMSE %
P01	Standard	40–44	–	218 ± 98	2.3 ± 1.6	5.6 ± 3.6
	Carousel	33	> 300*	> 300*	1.1 ± 0.4	3.4 ± 1.3
P02	Standard	32	–	> 150*	2.8 ± 0.7	8.6 ± 2.3
	Carousel	42	125 ± 37*	117 ± 31*	1.6 ± 0.4	3.9 ± 0.9
P03	Standard	45–50	–	43 ± 8	2.5 ± 1.4	5.1 ± 2.7
	Carousel	35–38	–	64 ± 11	3.7 ± 1.5	10.3 ± 4.6
P04	Standard	40	58 ± 16	61 ± 24	2.7 ± 1.1	6.8 ± 2.7
	Carousel	35–38	70 ± 16*	67 ± 21	3.6 ± 2.1	10.1 ± 6.2
P05	Standard	45–52	158 ± 38*	156 ± 41*	1.8 ± 0.9	3.8 ± 1.8
P06	Standard	25–50	83 ± 25	68 ± 22	2.3 ± 0.9	6.6 ± 2.1
P07	Standard	28–44	79 ± 27	76 ± 33	3.5 ± 2.0	10.5 ± 6.1

Lower RMSE and RMSE % indicates better tracking performance. Asterisks (*) indicate T_maxPW_ or T_target_ was not yet reached within the duration of one or more trials. In these instances, the maximum trial duration was used for outcome measure calculations. Dashes (–) indicate PW data unavailable

**Fig. 6:**
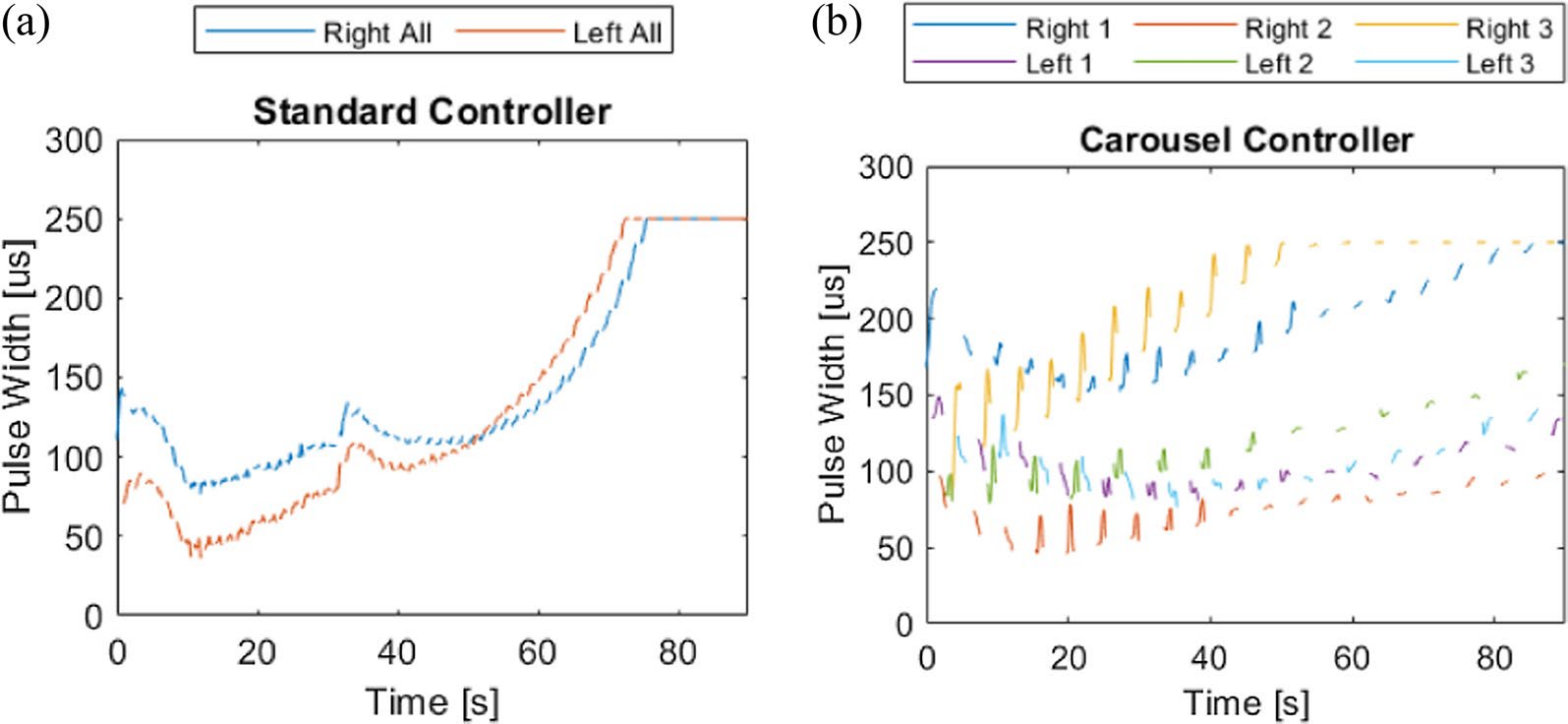
Example (**a**) S-Cont and (**b**) C-Cont PW output throughout a cycling trial for the same participant (P04). Colors indicate PWs delivered through independently-controlled electrode contacts. PW was adjusted as needed within each active contraction to maintain a target cadence, up to a hardware limited 255 µs maximum

Stimulus levels were dynamically adjusted below maximum PW values by the controllers to account for both muscle potentiation and fatigue (Fig. 6). Due to these adjustments by both controllers and the low duty cycle employed with C-Cont, Q accumulated less rapidly for controlled conditions relative to S-Max (Table 3). Lower total Q coupled with equal or greater W resulted in greater η for controlled conditions relative to S-Max (Table 3). Both standard and carousel controlled stimulation thus produced more work per unit charge injected than conventional stimulation.

**Table 3:** Work, charge accumulation, and calculated efficiency differences between controlled conditions and corresponding conventional stimulation trials

Participant	Controlled condition	Controlled mean W [kJ]	Controlled total Q [nC]	Controlled η [kJ/nC]	S-Max mean W [kJ]	S-Max total Q [nC]	S-Max η [kJ/nC]	Δη (× 10^–6^)
P01	Standard	2.94	–	–	2.75	–	–	–
	Carousel	4.51	1.24E6	3.64E−6	4.19	5.45E6	0.77E−6	+ 2.87
P02	Standard	0.96	–	–	1.14	–	–	–
	Carousel	3.15	4.81E5	6.55E−6	2.71	2.72E6	0.99E−6	+ 5.56
P03	Standard	1.62	–	–	1.61	–	–	–
	Carousel	1.33	–	–	1.18	–	–	–
P04	Standard	1.75	1.02E6	1.72E−6	1.52	1.63E6	0.93E−6	+ 0.79
	Carousel	1.69	3.48E5	4.86E−6	1.51	1.63E6	0.93E−6	+ 3.93
P05	Standard	5.57	5.87E5	9.49E−6	5.09	8.87E5	5.74E−6	+ 3.75
P06	Standard	2.67	5.17E5	5.16E−6	2.61	6.24E5	4.18E−6	+ 0.98
P07	Standard	1.58	3.97E5	3.97E−6	1.49	4.96E5	3.00E−6	+ 0.97

Dashes (–) indicate Q and η data unavailable. Positive Δη indicate superior efficiencies of controlled conditions relative to conventional stimulation

### Physiological outcomes: muscle oxygen saturation and heart rate

**Fig. 7:**
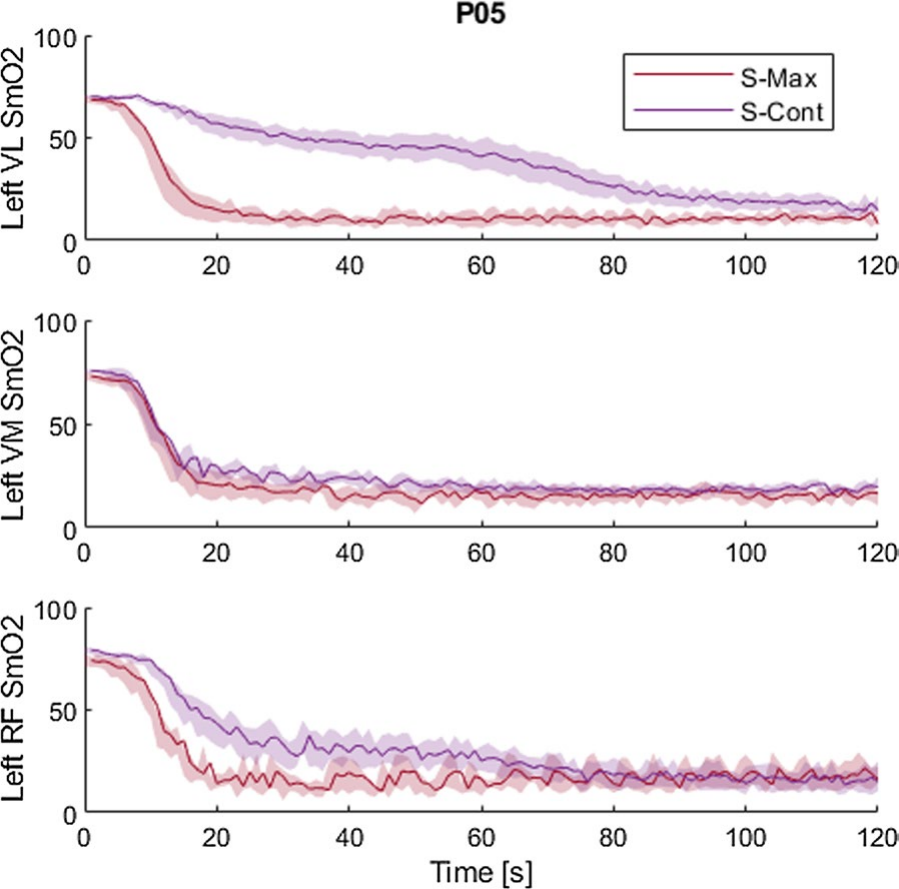
P05 muscle oxygenation (SmO_2_) over time in the left vastus lateralis (top), left vastus medialis (middle), and left rectus femoris (bottom) throughout cycling trials with S-Max (red) and S-Cont (purple) stimulation. Shaded regions represent standard deviations. Cadence control enables higher SmO_2_ values to be maintained in both the vastus lateralis and rectus femoris fiber groups during the first minute of exercise

**Fig. 8:**
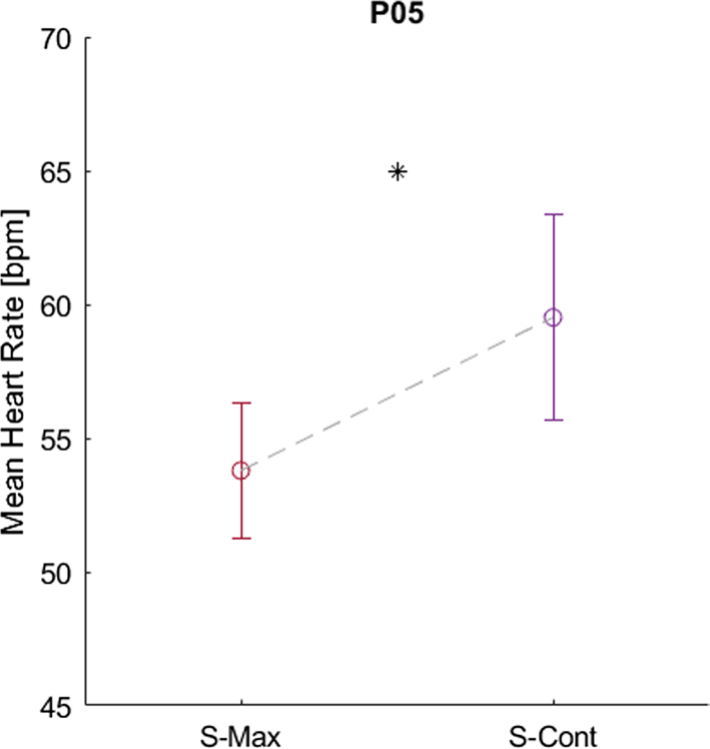
P05 average heart rate during S-Max and S-Cont stimulation-induced cycling bouts

Muscle oxygen saturation (SmO_2_) was measured with four participants (P04–P07) during S-Max and S-Cont cycling trials. No large declines in SmO_2_ or consistent differences in SmO_2_ trends between stimulation conditions were observed in three of the four participants (data not shown). In P05, however, S-Max induced large and rapid SmO_2_ declines in each head of the left quadriceps, from 75 to 80% at baseline to approximately 10% within the first 20 s of S-Max stimulation (Fig. 7). In contrast, S-Cont delayed SmO_2_ decline to similar levels until 75 and 120 s for the left rectus femoris and left vastus lateralis, respectively in that same participant (Fig. 7). P05’s average heart rate was also significantly higher during trials with S-Cont stimulation compared with trials using S-Max (59.5 ± 3.8 vs. 53.8 ± 2.5, p < 0.05) (Fig. 8).

## Discussion

The purpose of this study was to improve stimulation-induced cycling performance through feedback control of stimulus levels. We found that enforcing a submaximal but higher than typical steady-state exercise intensity through feedback control can significantly increase power maintained and work performed by participants with paralysis. We further found that cadence feedback control of stimulus levels reduced between- and within-stroke power fluctuations, enabling practical implementation of duty cycle reducing paradigms that can further extend exercise durations prior to fatigue. These results may be applied in the development of enhanced stimulation systems and the formation of optimal exercise training regimens for participants with paralysis.

### Cycling endurance and intensity

Cadence-controlled stimulation significantly improved endurance in six out of seven participants, as evident by higher P_end_ values maintained by at least one controlled condition. Participants (P03, P04) who showed remarkable improvements (~ 180% to ~ 900%) in P_end_ with cadence-control initially output high peak powers but often could not cycle 90 s continuously with S-Max, resulting in near zero P_end_ values. Both controlled conditions consistently enabled them to maintain power throughout their entire 90 s trial durations, leading to the large percent increases. For participants who produce lower initial peak powers (P02, P06), P_end_ improvements with cadence control were not always significant. Training should first focus on increasing absolute strength to support cycling against meaningful resistances for these participants. Enabling users to extend exercise durations prior to lower extremity muscular fatigue by increasing baseline strength and/or applying closed-loop control of stimulation will ultimately increase benefits to those muscles and provide a better cardiovascular workout.

Chosen target cadences demanded power outputs greater than a participant’s typical steady state ability and could not always be sustained throughout an entire trial duration. Nevertheless, even when controllers reached maximal stimulus levels and were unable to recruit any additional fibers to maintain a target cadence, end powers still consistently remained above those of conventional stimulation. Cadence control eliminated the high initial peak powers that occur with conventional stimulation by recruiting fewer fibers with submaximal stimulus levels. Submaximal stimulus levels avoid electric field overlap and can prevent fibers that would be in the overlapped regions from experiencing excitation–contraction decoupling due to unintentionally high firing frequencies. Fewer fibers initially contracting can cause a lower accumulation of hydrogen ions and other metabolic byproducts that contribute to force decline. Together these potential mechanisms may account for the improvements in end power achieved with the controllers.

Total work performed within a set cycling duration was also strongly affected by target cadence choice and the resultant power output. We could not ensure a priori that a chosen target output could be maintained long enough to produce equal or greater work within the same cycling duration. Still, the fact that work did accumulate to a significantly greater degree in three participants is encouraging. Extending trial durations would likely further increase differences in total work between S-Max and both controlled conditions for all participants.

Consistently performing more work and maintaining higher steady state powers within each exercise session is crucial to maximizing physiological benefits. Competitive able-bodied cyclists track a functional threshold power (FTP), the maximum steady state power they can maintain for 1 h [22]. Training programs that vary workout durations and intensities based on % FTP have been shown to significantly improve FTP and provide physiological benefits for recreational and competitive cyclists [23]. Such FTP measurements and fine-tuned programs have historically been unavailable to persons with paralysis, as conventional stimulation systems do not sustain a steady muscle power output. The cadence-controlled stimulation schemes presented here could now be used to establish and track a modified FTP in this population. Long-term training regimens based on the FTP equivalent for stimulated cycling could be explored in future studies.

### Power fluctuation and controller performances

Uneven force production among independently activated fiber groups was a significant practical limitation of duty cycle reducing stimulation patterns in prior studies [13, 24]. In this study, the C-Cont pattern successfully reduced PFI to the point of no significant difference with S-Max in three of four tested participants and to significantly lower values than both S-Max and S-Cont in the fourth participant. PFI improvements over S-Max are likely due to controllers enforcing more even outputs between the left and right legs, while C-Cont improvements over S-Cont may be due to more precise adjustments by each contact’s individual PI controller. S-Cont modulates and delivers the same PW through all active knee extensor contacts at once, thus operating along a combined recruitment curve (RC) from all activated MUPs. Outputs from independent MUPs activated at the same time will sum approximately linearly when stimulated fiber overlap is low [25, 26]. The combined RC then is likely very steep, especially at lower stimulus values. Small changes in PW could result in large relative changes in muscle output, and the S-Cont controller may have not been as well tuned to account for this, resulting in higher PFIs and RMSE than C-Cont. Conversely, C-Cont modulates PW through the single active knee extensor contact with its own dedicated PI controller. Adjustments in PW thus only depend on what the one knee-extensor MUP needs to meet the desired cadence, removing the potential of over- or under-stimulating through other simultaneously active contacts as is possible with S-Cont. This study thus demonstrates a practical way to employ duty cycle reducing stimulation patterns to better improve cycling ability without the limitations of open-loop implementation.

From the available in-laboratory data, cadence-controlled patterns were more efficient than conventional stimulation, producing more work per unit of charge injected. Efficiency even increased when work was not significantly higher, due to lower levels of Q needed to sustain the target output. Higher efficiency may extend battery life of the stimulation systems and provide further assurance that no overstimulation or damage to the neural tissue will result over time, as similar outputs may be achieved with less injected charge [27]. Additionally, prior research shows a correlation between stimulation cost, the inverse of efficiency presented here, and the oxygen cost (the rate of pulmonary oxygen uptake) of an exercise for a given power output [19]. Greater stimulation efficiency with cadence-control may therefore coincide with less oxygen cost, which could make maintaining a greater exercise intensity more feasible for deconditioned persons who may have partial paralysis of the ventilatory muscles. Though not formally measured in this study, P05 anecdotally reported less breathlessness after controlled cycling bouts compared with conventional stimulation.

Cadence only steadily declined below target once controllers were unable to recruit more unfatigued MUPs. For P04, the only participant for whom PW data from both controlled conditions is available, both T_maxPW_ and T_target_ occur later with C-Cont than S-Cont for comparable target outputs. All contacts required maximum PWs by the end of each S-Cont trial, while four of the six independently controlled contacts do not yet reach maximum with C-Cont (Fig. 6). This agrees with our prior open-loop study [13] where reduced duty cycles were found to further extend muscle output. By incorporating reduced duty cycles into closed-loop stimulation models, originally recruited fibers fatigue less rapidly and additional fibers do not need to be recruited as frequently, enabling the controller to work below maximum PWs and sustain target output even longer. This also supports the assumption that independent fiber groups are activated by submaximal PWs through independent contacts with low overlap, and are thus able to periodically rest during carousel stimulation, despite lacking formal overlap assessments in this participant.

Both controllers only adjusted stimulation through contacts that activated the knee extensors. Stimulation to the knee flexors, hip extensors, and adductors remained constant at maximal values. Progressive fatigue in these other muscle groups may have influenced controller adjustments, potentially resulting in premature increases in quadriceps activation to make up for declining output of the other muscles. Despite this limitation, basic PI control of only the knee extensor fiber groups yielded average absolute RMSE across all participants of only 2.4 rpm (6.4%). Controlling stimulus levels to all involved muscle groups may further improve target tracking ability and would likely increase the performance benefits of S-Cont and C-Cont stimulation. While direct comparisons to other fatigue-delaying strategies are not made, improvements achieved in this study may also be further enhanced by incorporating asynchronous, interleaved, or other such strategies [14–16, 28] for all involved muscle groups during closed-loop cycling. More sophisticated control schemes have been proposed from simulations with musculoskeletal models to optimize performance and more faithfully produce the desired output [18, 29, 30]. However, these proposed control schemes have not yet been successfully deployed in clinical tests with paralyzed participants or without simultaneous, prioritized control of a motor. Future work should seek to incorporate these more advanced controllers into a motorless stimulation system to potentially provide even greater improvements in exercise performance and physiological outcomes.

Recently, a closed-loop control scheme using feedback from IMU sensors to spatiotemporally adjust implanted epidural electrical stimulation was shown to improve power output during cycling in one participant with SCI [31]. The study’s control scheme focused on adjusting the timing and location of epidural stimulation, not on regulating the intensity of resulting muscle contractions for cadence control. The study also only presents data for less than 75 pedal strokes, fewer than what was accomplished in most of the trials reported herein, and it is unclear if the participant could have continued further with this strategy. It is thus difficult to compare the effectiveness of these closed-loop, implanted strategies, but it is probable that the epidural modality would also benefit from similar cadence-control techniques.

### Physiological effects of controlled stimulation

Muscle oxygenation data may explain variations in functional performance among select participants. In P05, S-Cont delayed rapid declines in SmO_2_ and resulted in significant functional improvements compared with S-Max. In the other participants tested, however, such drastic SmO_2_ declines did not occur with any stimulation pattern. P05 may have had a previously undetected perfusion issue that limited oxygen delivery to the working muscles relative to their oxygen consumption. By delaying the incorporation of some fibers until needed with a controller, oxygen was not depleted immediately in all fibers. This may enable more efficient aerobic respiration to occur in some parts of the muscles longer than with S-Max, and account for the significant improvements in cycling performance. P05 also cycled against greater resistances and produced much greater powers than the other participants, which contributes to more dramatic declines in SmO_2_ [32] and provides ample opportunity for improvement with the controller. It is possible that, once other participants gain strength to cycle against greater resistances, the controllers will become more beneficial as demand on the muscles increases and better pacing strategies become more valuable. Future work should assess the relative rates of SmO_2_ decline at various gears and cadences to determine a combination that enables high power outputs with the physiological advantage of slower oxygenation decline.

A significant heart rate increase when cycling with cadence-controlled stimulation is another notable physiological benefit seen in P05. Paralysis, particularly when caused by SCI, hinders appropriate cardiorespiratory responses to stimulated exercise [33]. Loss of quick afferent feedback from the working muscles to the autonomic nervous system due to the spinal cord lesion eliminates the influence of the exercise pressor reflex on the regulation of heart rate, respiration rate, and blood pressure during exercise. We have observed heart rate often changes only negligibly and sometimes even declines in participants with SCI despite cycling to the point of lower extremity exhaustion. The reduction in heart rate is likely due to increased venous return when the typically sedentary lower extremities are activated by stimulation. Specifically, contraction of the paralyzed muscles creates a pumping effect that can greatly increase venous return and stroke volume and subsequently reduce heart rate for any given cardiac output. These factors prevent conventional stimulation-induced cycling from providing a meaningful cardiovascular workout and present obstacles for providing the working muscles with the resources needed to keep moving. The ability of cadence-controlled stimulation to overcome these barriers to elevating heart rate in P05 is extremely promising. Greater work achieved with the controller may explain the elevated heart rate in this participant. Additionally, altered hemodynamics may have also played a role. The controller maintains a submaximal cadence and does not maximally recruit all available fibers from the outset of the exercise, causing only a subsection of the quadriceps to contract and relax at comparatively slower rates. Using only a subsection of available fibers to maintain a submaximal cadence may reduce venous return through decrease muscle pump compared with conventional stimulation that activates all available fibers at higher cadences [34]. This could potentially ease the blood volume-induced heart rate depression, further contributing to the greater heart rates achieved. The statistically significant increase in heart rate, while only 6 beats per minute, may have facilitated greater oxygen delivery to the quadriceps muscles to help P05 sustain power and perform even more work, perpetuating the cycle.

### Advantages of motorless exercise control

Prior studies incorporating feedback control during stimulation-induced cycling utilized a motor to assist or resist stimulated muscle contractions to maintain a target cadence and/or power output [18, 35–38]. These approaches can provide greater resistance in the beginning of a trial to maximize the load against which the muscle must work before it fatigues. Maximizing resistive loads may be the key to achieving greater load-dependent physiologic improvements with these systems, especially in bone density [39, 40]. However, excessive motor resistance may prematurely fatigue the muscles and drastically reduce the duration of exercise. Conversely, assisting the pedaling motion when muscle output becomes insufficient for target maintenance can shield paralyzed musculature from positive stress and decrease required effort that would help them improve. There are mixed opinions as to whether keeping the legs cycling after the muscles can no longer contribute to the motion provides adequate physiological benefits [41], and the fatigued muscles may be better served resting without continued ineffective stimulation so that they may recover and perform subsequent bouts of meaningful, leg-driven (as opposed to motor-driven) cycling. Additionally, integrating a motor significantly increases the complexity of the control algorithm since care must be taken not to risk harming the participant, and increases the weight and cost of the cycling apparatus. Control methods from this study are easily implemented with no additional hardware or musculoskeletal modeling requirements and only minute increases in computational complexity, making them ideal for practical every day and potentially overground use. Maintaining a mid-level intensity using only the capabilities of the activated muscles may enable meaningful work against resistive loads without undue fatigue or motorized assistance. Future work should determine the relative advantages of the simple control strategies presented here compared to those that employ motors.

## Conclusions

Cadence control of neural stimulation intensity successfully extended cycling endurance in a motorless system, supporting our hypothesis. Both standard and duty cycle reducing cadence control schemes were found to prolong activated muscle output compared with conventional stimulation techniques by maintaining a mid-level exercise intensity. Extending exercise durations without interference of a motor will allow participants with paralysis to obtain greater physiological benefits, as demonstrated by preliminary heart rate and muscle oxygen saturation measurements that improved significantly with cadence control. Significantly higher power maintenance at the end of controlled trials may enable more work to accumulate with increased trial durations, thus increasing the intensity and potential physiological benefits of exercise. Finally, simple control schemes used in this study provided stable power output and good target cadence tracking performance with minimal processing and no training or modeling required, making them suitable for widespread, practical use with the potential to enable overground cycling. While this study used implanted stimulation, surface-based systems capable of eliciting sufficiently distinct and graded responses from targeted muscles may also benefit from these simple control schemes. Future longitudinal studies to track the long-term effects of training with cadence-controlled neural stimulation are warranted. Future work may also seek to combine these closed-loop approaches with other strategies that have shown promise for delaying fatigue, such as interleaved stimulation.

## Data Availability

The datasets analyzed in the current study are available from the corresponding author on reasonable request. Direct correspondence to kxg277@case.edu.

## Abbreviations

SCI: Spinal cord injury
MUP: Motor unit pool
ECU: External control unit
S-Max: Standard maximum stimulation
S-Cont: Standard controlled stimulation
PW: Pulse width
C-Cont: Carousel controlled stimulation
PI: Proportional, integral control
W: Work
P_end_: End power
PFI: Power fluctuation index
RMSE: Root mean squared error
T_target_: Time on target
Tmax PW: Time to maximum PW
Q: Charge
PA: Pulse amplitude
η: Efficiency
SmO_2_: Muscle oxygenation
FTP: Functional power threshold
RC: Recruitment curve
